# Heptazine-Based *π*-Conjugated Materials for Light-Emitting

**DOI:** 10.3389/fchem.2021.717569

**Published:** 2021-06-18

**Authors:** Jie Li, Li Tao, Yanqing Wang, Yali Yao, Qiang Guo

**Affiliations:** ^1^ College of Optoelectronic Technology, Chengdu University of Information Technology, Chengdu, China; ^2^ College of Polymer Science and Engineering, Sichuan University, Chengdu, China; ^3^ School of Physical Education, Chengdu Normal University, Chengdu, China

**Keywords:** heptazine, light-emitting, metal ion-containing heptazine, polymeric heptazine, monomeric heptazine

## Abstract

On the basis of planar and relatively rigid nitrogen-rich heterocyclic system of the heptazine core, heptazine-based *π*-conjugated materials have aroused widespread attention over the past decade by virtue of the fascinating electronic, optical, thermal, and mechanical properties in the fields of light-emitting, photocatalysis, sensors, environmental remediation, and so forth. However, there are still several obstacles to be solved before practical applications, such as low photoluminescence quantum efficiencies for light-emitting and weak visible absorption for photocatalysis. To further enhance various properties of heptazine-based *π*-conjugated materials, a series of strategies have been developed, including ingenious molecular design and modification, novel synthetic, and preparation methods. In this review, the significant progress of monomeric and polymeric heptazine-based *π*-conjugated materials and their applications typically in light-emitting are reviewed, which is beneficial for the acceleration of practical applications of heptazine-based materials and devices.

## Introduction

Over the last decade, considerable progress in the fields of organic light-emitting diodes (OLEDs) and photocatalysis has triggered intensive effort to develop highly efficient light-emitting materials and photocatalysts (Wu and Ma, 2016; Zhou et al., 2017; Zhou et al., 2019; Yin et al., 2020; Zhou et al., 2020). Among the many kinds of materials investigated, nitrogen-rich heptazine-based materials are highly attractive on account of intriguing photoelectronic and photocatalytic properties (Audebert et al., 2021; Wang et al., 2021). In the 1830s, heptazine-based materials were initially discovered by a Swedish chemist, Jons Jakob Berzelius, after the ignition of mercury thiocyanate, and this work was mentioned and reported by the German chemists, Justus von Liebg and Leopold Gmelin (Liebig, 1834; Gmelin, 1835; Liebig, 1835). Meanwhile, the compound obtained by Berzelius was termed melon by Liebig. However, the study on heptazine derivatives has long been hampered probably by their general insolubility, chemical inertness, and high decomposition temperatures which make their characterization and modification difficult (Kailasam et al., 2013; Sayed et al., 2017). About 100 years later, through the elaborate analysis of a few small crystals by X-ray crystallography, Pauling, and Sturdivant proposed a planar triangular structure as the basic monomer of melon, cyameluric nucleus (C_6_N_7_), which is the accurate structure of heptazine core (Pauling and Sturdivant, 1937). Much later, the unsubstituted heptazine, 1,3,4,6,7,9,9b-heptaazaphenalene (C_6_N_7_H_3_), was firstly synthesized and characterized in the 1980s by the members of Leonard group (Hosmane et al., 1982; Shahbaz et al., 1984). Since 2001, the structure of Berzelius’s melon has been systematically confirmed, which is polymerized with the heptazine units linked through an amine (NH) link (Komatsu, 2001; Lotsch et al., 2007).

**FIGURE 1:**
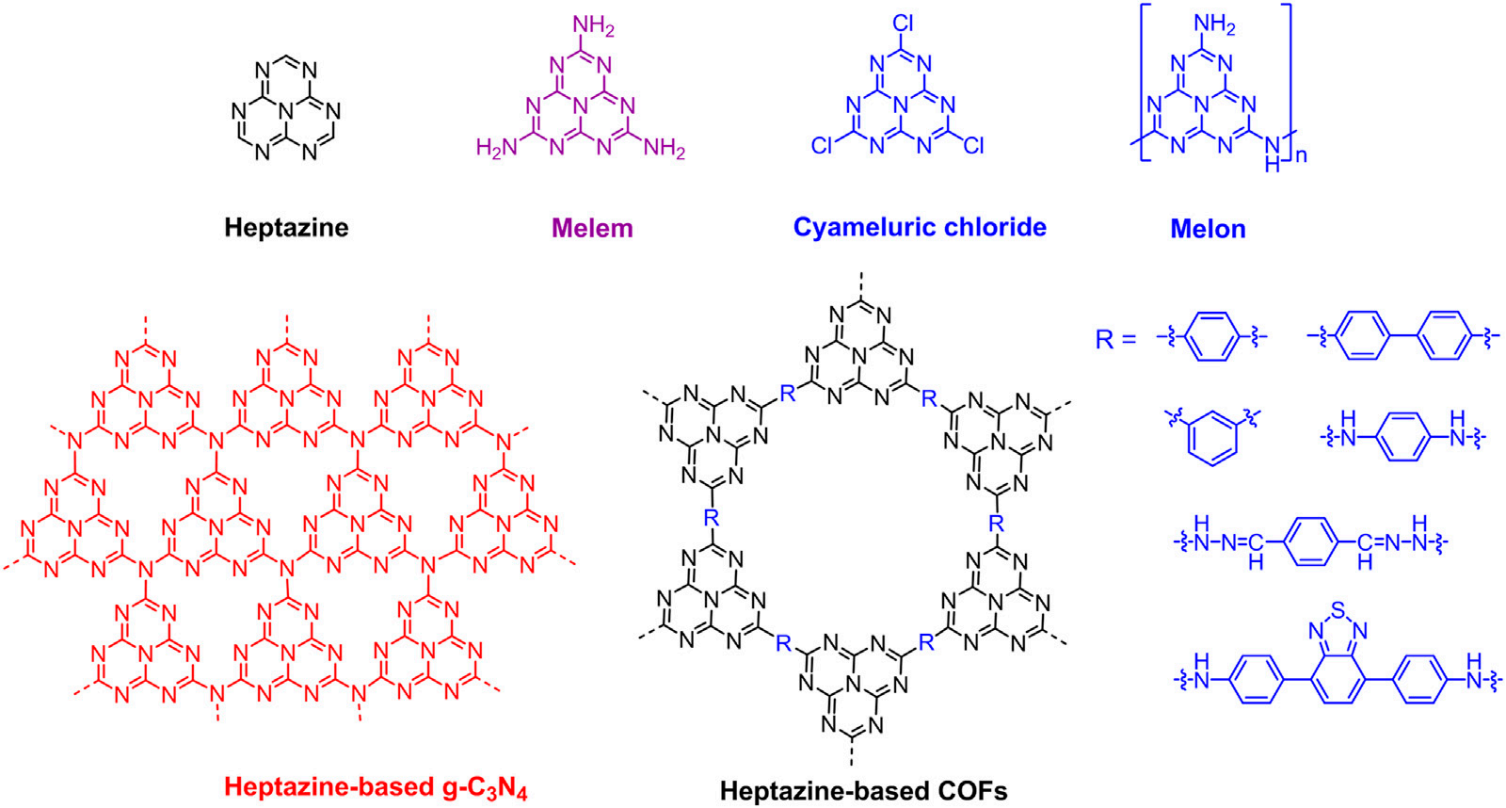
Chemical structures of heptazine, melem, cyameluric chloride, melon, heptazine-based graphitic carbon nitride (g-C_3_N_4_), and covalent organic frameworks (COFs).

The heptazine, also known as tri-*s*-triazine, carbon nitride or C_6_N_7_, is a type of chemical compound consisting of a triangular structure, or three fused *s*-triazine rings, with three substituents at the corners of the triangle. Namely, the heptazine is a planar and relatively rigid nitrogen-rich heterocyclic system with 6 C=N bonds surrounding an sp^2^-hybridized N atom. The heptazine with three amino substituents is called melem (2,5,8-triamino-tri-*s*-triazine), which is an important intermediate during condensation of melamine to graphitic carbon nitride (g-C_3_N_4_). Jurgens et al. initially ascertained the crystal structure of melem by X-ray powder diffractometry and found that melem molecules are arranged into parallel layers with an interplanar distance of 0.327 nm. Particularly, according to temperature-dependent X-ray powder diffractometry investigations above 560°C, they discovered that the melem would transform into g-C_3_N_4_ (Jurgens et al., 2003). Similar to cyanuric chloride (trichloro-*s*-triazine, C_3_N_3_Cl_3_), cyameluric chloride (trichloro-tri-*s*-triazine, C_6_N_7_Cl_3_) is an important starting material for various synthesis of heptazine-based materials, and Kroke et al. comprehensively characterized its crystal structure and photophysical properties (Kroke et al., 2002). As the heptazine-based g-C_3_N_4_ emerging to be a class of promising metal-free photocatalysts, it received tremendous research interests over the past decade in the fields of hydrogen evolution Wang et al. (2009), Liao et al. (2019), CO_2_ reduction Gao et al. (2016), Barrio et al. (2019), photocatalytic degradation of organic pollutants Ong et al. (2016), Zeng Y. et al. (2018), and artificial photosynthesis (Su et al., 2010; Dai et al., 2018). Additionally, heptazine-based covalent organic frameworks (COFs) have also attracted much attention in the past several years due to the photocatalytic performance (Bojdys et al., 2010; Kailasam et al., 2016; Luo et al., 2019; Xing et al., 2020; Zhang et al., 2020). The various applications are significantly associated with the appealing heptazine-based molecular structure in which the sp^2^ hybridized carbon and nitrogen induce a delocalized *π*-conjugated system and consequently result in a moderate band gap of around 2.7 eV, whereby a broad variety of photocatalytic reactions can be carried out (Zhang et al., 2019; Patnaik et al., 2021). The chemical structures of heptazine, melem, cyameluric chloride, melon, heptazine-based g-C_3_N_4_, and COFs are depicted in Figure 1. Considering a number of published review articles with respect to the photocatalysis of g-C_3_N_4_ and in order to avoid the content overlap, in this article, the heptazine-based materials regarding to light-emitting are mainly reviewed.

## Metal Ion-Containing Heptazine-Based Light-Emitting

In 2012, Makowski et al. synthesized a series of rare-earth melonates LnC_6_N_7_(NCN)_3_•*x*H_2_O (Ln = La, Ce, Pr, Nd, Sm, Eu, Tb; *x* = 8–12) by metathesis reactions in aqueous solution and systematically investigated the photoluminescence (PL) performance of LnC_6_N_7_(NCN)_3_•*x*H_2_O (Ln = Eu, Tb; *x* = 9–12). The terbium melonate showed green emission with an emission peak (*λ*_em_) of 545 nm due to the ^5^D_4_→^7^F_5_ transition. Meanwhile, they found that the rare-earth melonates exhibited rather low thermal stability probably deriving from the tight binding of crystal water, which resulted in hydrolytic decomposition at elevated temperatures (Makowski et al., 2012). Cheng et al. reported a silver-g-C_3_N_4_ quantum dots (Ag-g-CNQDs) composite prepared from g-C_3_N_4_ quantum dots and silver nanoparticles by water phase synthesis (Cheng et al., 2018). Based on metal-enhanced fluorescence, the Ag-g-CNQDs composite exhibited excitation-dependent red emission with *λ*_em_ = 600 nm and a PL quantum efficiency (PLQE) of 21%. More importantly, for heparin detection, the emission at 600 nm was enhanced linearly over a concentration range of 0.025–2.5 μM by hydrogen-bonding and electrostatic interaction. This phenomenon has also been successfully applied to determine heparin levels in human serum samples, demonstrating its promising diagnostic applications.

## Polymeric g-C_3_N_4_-Based Light-Emitting

As an organic semiconducting material, g-C_3_N_4_ has drawn immense attraction due to its unique optical and electronic properties. In 2008, Iwano et al. studied the possibility for white light emitting devices using carbon nitride thin films prepared by microwave electron cyclotron resonance (ECR)-plasma chemical vapor deposition (CVD). The cathodoluminescence measurement of the film grown by ECR-plasma CVD method showed three peaks of red, green, and blue (Iwano et al., 2008). Barman et al. presented highly blue fluorescent g-C_3_N_4_ quantum dots (g-CNQDs) with a PLQE of 29% (Barman and Sadhukhan, 2012). Notably, the g-CNQDs can play a dual role for selective and sensitive detection of mercuric ions and iodide ions in aqueous media by “ON-OFF-ON” fluorescence response. Zhou et al. reported a low-temperature solid-phase method to synthesize highly fluorescent g-CNQDs with a PLQE of 42% (Zhou et al., 2013). Interestingly, the emission of g-CNQDs can be tuned from deep blue to green by adjusting the molar ratio of the two reactants, urea and sodium citrate.

Zhang et al. facilely synthesized g-C_3_N_4_ by the low temperature thermal condensation of melamine between 300–650°C and found that the PL spectra could be continuously tuned from 400 to 510 nm with the control of temperature (Zhang Y. et al., 2013). Chen el al. characterized the fluorescence and electrochemiluminescence (ECL) properties of g-C_3_N_4_ nanoflake particles (g-C_3_N_4_ NFPs) and nanoflake films (g-C_3_N_4_ NFFs). It was found that both g-C_3_N_4_ NFPs and g-C_3_N_4_ NFFs showed strong blue emission, and the as-prepared g-C_3_N_4_ NFFs exhibited strong non-surface state ECL activity in the presence of reductive-oxidative coreactants with *λ*_em_ = 435 nm (Chen et al., 2013). Two-dimensional nanosheets have attracted tremendous attention because the atomic-thick nanosheets can not only enhance the intrinsic properties of their bulk counterparts but also generate new promising properties. In 2013, Zhang et al. firstly prepared ultrathin g-C_3_N_4_ nanosheets by a liquid exfoliation route from bulk g-C_3_N_4_ in water (Zhang X. et al., 2013). In comparison to the bulk g-C_3_N_4_, the ultrathin blue-emitting g-C_3_N_4_ nanosheets showed enhanced intrinsic photoabsorption and photoresponse, resulting in an extremely high PLQE of 19.6%, which could be promising candidates for bioimaging application.

To investigate the charge carrier trapping, migration, and transfer of electron-hole pairs, Zhao et al. synthesized a series of g-C_3_N_4_ under different precursor masses and measured their PL characteristics. All the as-prepared g-C_3_N_4_ samples showed blue emission with *λ*_em_ = 440–455 nm. Moreover, CN-2T exhibits the highest PL intensity, which is attributed to the less structural defects (e.g. uncondensed −NH_2_, −NH groups) in view of more complete condensation of thiourea (Zhao et al., 2015). To better clarify the photocatalytic mechanism of heptazine-based materials, Wen et al. measured the photophysical properties of melamine, melem, and g-C_3_N_4_. They found that the PL intensities of melem is the highest, g-C_3_N_4_ second, and melamine the weakest, indicating that the condensation of melamine to melem makes PL stronger, while the condensation of melem to g-C_3_N_4_ results in weaker PL (Wen et al., 2018). Yang et al. prepared strong blue-emitting porous g-C_3_N_4_ with *λ*_em_ = 400 nm and a PLQE of 21% (Yang et al., 2018). Compared with bulk g-C_3_N_4_ and g-C_3_N_4_ nanosheets, the porous g-C_3_N_4_ shows good PLQE, large surface area and great dispersibility, and stability in water. The porous g-C_3_N_4_ probes showed the remarkable sensitivity and selectivity for uric acid (UA) and were successfully applied to the determination of UA.

Recently, Yadav et al. developed a facile approach to prepare free-standing films comprising of g-C3N4 nanolayers (Yadav et al., 2020). The as-synthesized g-C3N4 film exhibited intense and broad blue emission centered 459 nm. Tang el al. realized the broadband white light luminescence based on electron-deficient porous g-C_3_N_4_ constructed by supramolecular copolymerization design (Tang et al., 2020). Meanwhile, they successfully narrowed the band gap of g-C3N4 from 2.64 to 1.39 eV. Furthermore, the emission wavelengths of electron-deficient porous g-C3N4 can be tuned from narrow blue to broad-band white range by the addition of 2, 4, 6-triaminopyrimidine (TAP).

## Monomeric Heptazine-Based Light-Emitting

### Traditional Fluorescence

Owing to the strong electron-withdrawing ability and three substitution sites at the corners of the triangle of heptazine core, a series of heptazine derivatives have been developed and the PL or electroluminescence (EL) characteristics have been investigated. During the structure determination of cyameluric chloride and melem, their photophysical properties were also measured. Cyameluric chloride showed blue emission with *λ*_em_ = 466 nm, and melem exhibited ultraviolet emission with *λ*_em_ = 366 nm and a relatively high PLQE of 40% (Kroke et al., 2002; Jurgens et al., 2003). In 2017, Zheng et al. reported a rod-like structured blue-emitting melem which was synthesized by treating a bulk melem with nitric acid and ethylene glycol (Zheng et al., 2017). Excitingly, the PLQE of the rod-like melem (56.9%) is about 1.6 times higher than that of the ordinary melem (35.2%) and is substantially higher than that of blue-emitting bulk g-C_3_N_4_ (4.8%). This blue-emitting melem shows great potential for practical applications in many fields.

**FIGURE 2:**
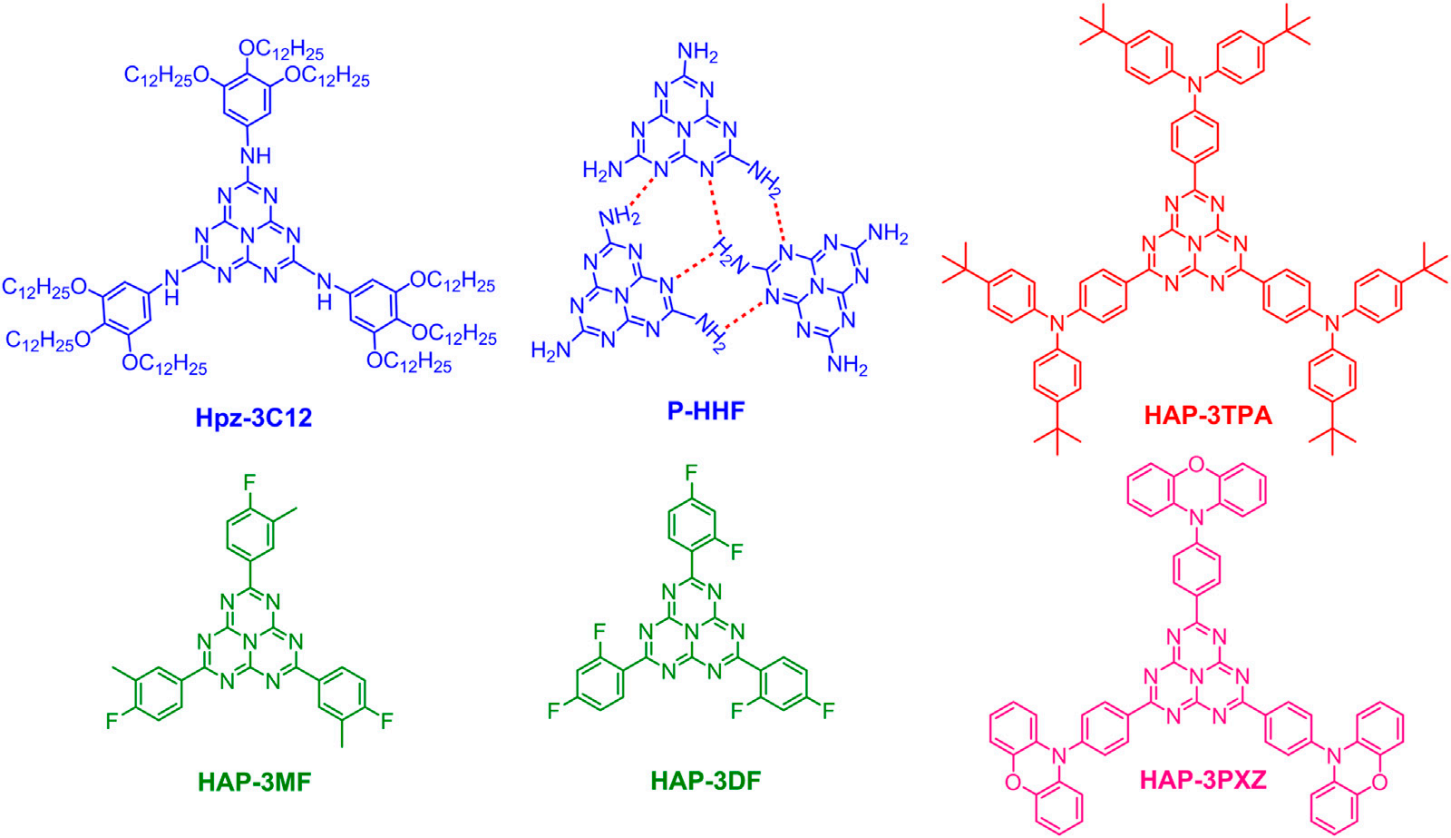
Chemical structures of Hpz-3C12, polymeric hydrogen-bonded heptazine frameworks (P-HHF), HAP-3TPA, HAP-3MF, HAP-3DF, and HAP-3PXZ.

Bala et al. reported a heptazine-based discotic liquid crystal molecule (Hpz-3C12, Figure 2), which presented significant aggregation-induced emission (AIE) behavior as indicated by the remarkably increased fluorescence intensity in solid state in comparison to that in solution (Bala et al., 2018). Interestingly, the EL spectra of solution-processed OLEDs containing Hpz-3C12 varied with the host materials. The OLED incorporating 3 wt% Hpz-3C12:CBP exhibited the best performance with a power efficiency of 0.3 lm W^−1^, a current efficiency of 0.4 cd A^−1^, an EQE of 1.6% and deep blue emission. Although the EL efficiency is low, it still demonstrates that heptazine-based discotic liquid crystals may contribute to the further development of AIE-based blue emitters. Yang et al. investigated the hydrogen-bonding effect on PLQEs and luminescence stability of polymeric hydrogen-bonded heptazine frameworks (P-HHF, Figure 2) and trivalent europium ions incorporated P-HHF (P-HHF-Eu) (Yang et al., 2020). The hydration degrees and the role of hydrogen bonding in the emission properties were analyzed by time-resolved and steady state PL spectroscopies. They found that the bulk P-HHF particles showed blue emission and a moderate PLQE of 35.8%, while enhanced PLQE of 55.9% was obtained when suspending P-HHF into polyvinyl alcohol (PVA) to form hydrogel composites (P-HHF-PVA gel). This work is considerably beneficial to understand the effect of intermolecular hydrogen-bonds on the luminescence characteristics of heptazine-based materials.

### Thermally Activated Delayed Fluorescence

Over the past decade, as the third generation organic light-emitting materials with respect to traditional fluorescent and phosphorescent materials, thermally activated delayed fluorescence (TADF) materials exhibit great potential in OLEDs on account of the excellent performance with both high EL efficiency and low cost (Uoyama et al., 2012; Zhang et al., 2014; Sohn et al., 2020). The key design strategy of TADF materials is to realize a small energy gap (Δ*E*_ST_) between the lowest excited singlet (S_1_) and triplet (T_1_) states through an ingenious design to effectively separate the electron densities of the highest occupied molecular orbital (HOMO) and the lowest unoccupied molecular orbital (LUMO) (Endo et al., 2011). One of the most widely used methods in developing highly efficient TADF emitters is to design molecules possessing electron donor-acceptor (DA) structure which is beneficial for the realization of small singlet-triplet splitting during intramolecular charge-transfer (CT) transitions (Zhang et al., 2012; Li et al., 2013; Tanaka et al., 2013; Kawasumi et al., 2015; Chen et al., 2018; Zeng W. X. et al., 2018). Based on the strong electron-withdrawing ability of heptazine core and strong electron-donating feature of triphenylamine, Li et al. designed and synthesized a highly efficient heptazine-based TADF emitter, HAP-3TPA (Figure 2), which exhibited a small Δ*E*_ST_ of 0.27 eV based on density functional theory (DFT) in view of the effective separation of HOMO and LUMO. Meanwhile, HAP-3TPA showed relatively high thermal stability over 500^o^C and considerably strong absorption centered at 486 nm. Encouragingly, an extremely high PLQE of 91%, red emission with *λ*_em_ = 610 nm were achieved in a 6 wt% HAP-3TPA:2,6-dicarbazolo-1,5-pyridine (26 mCPy) doped film. More importantly, An OLED incorporating 6 wt% HAP-3TPA:26 mCPy as an emitting layer exhibited a fairly high external quantum efficiency (EQE) of 17.5 ± 1.3%, up to now, which is still one of the highest EQEs of red TADF emitters (Li et al., 2013). As a result, the realization of highly efficient red emitter makes TADF completely cover the red, green and blue bands, and show promising applications in the fields of lighting and display.

Alternatively, Goushi et al. demonstrated that small Δ*E*_ST_ can be realized by exciplex formation *via* intermolecular CT between two molecules with electron-donating and electron-accepting characteristics, respectively (Goushi et al., 2012). Since then, a new class of TADF emitters have been developed (Chapran et al., 2019; Wu et al., 2019; Zhang et al., 2021). In view of the formation mechanism of exciplex, another heptazine derivative (HAP-3MF, Figure 2) was designed and synthesized, and an exciplex system was formed by choosing 1,3-di (9*H*-carbazol-9-yl)benzene (mCP) as an electron donor (Li et al., 2014a). Surprisingly, the 8 wt% HAP-3MF:mCP doped film showed efficient exciplex emission with a remarkably high PLQE of 66.1%, a rather small PLQE difference was observed in air and vacuum conditions, indicating the tight molecular packing between HAP-3MF and mCP. Subsequently, the OLED containing 8 wt% HAP-3MF:mCP showed a pretty high EQE of 11.3% with a low roll-off, demonstrating the efficient harvest of triple exciplex excitons through reverse intersystem crossing (RISC) from T_1_ to S_1_ under electrical excitation. By changing the three substituents of heptazine core from 2-fluorotoluene to 1,3-difluorobenzene, 2,5,8-tris(2,4-difluorophenyl)-1,3,4,6,7,9,9b-heptaazaphenalene (HAP-3DF, Figure 2) was obtained (Li et al., 2021). The OLED incorporating 8 wt% HAP-3DF:mCP as an emitting layer exhibited a reasonably high EQE of 10.8%.

Additionally, TADF can be also realized by more localized n→π* transitions involving the nonbonding lone-pair electrons of heteroatoms and *π* antibonding molecular orbitals (Turro et al., 2010). Excitingly, through elaborate theoretical analysis and experimental verification, a hidden, efficient TADF pathway was found in HAP-3MF on account of n→π* transitions (Li et al., 2014b). To verify the contribution of n→π* transitions and prevent exciplex formation, electron-deficient bis[2-(diphenylphosphino)phenyl] ether oxide (DPEPO) was chosen as the host material. An OLED incorporating 6 wt% HAP-3MF:DPEPO showed a high EQE of 6.0% regarding to the low PLQE of 26%, confirming the hidden n→π* based TADF pathway. This work demonstrated that the n→π* emitter is a new TADF material and can be applied to OLED applications. Interestingly, the n→π* based HAP-3DF exhibited a lower PLQE of 0.16 and an EQE of 3.0%, illustrating that the subtle structural change has a great influence on luminescence properties (Li et al., 2021). According to the energy gap law, the design of efficient red-emitting materials is rather difficult. In 2018, Kang, et al. proposed an efficient heptazine-based red TADF molecule, HAP-3PXZ (Figure 2), based on the optimal Hartree-Fock percentage calculation method through enlarging the delocalization of HOMO and LUMO (Kang et al., 2018). Excitingly, HAP-PXZ exhibited deep red emission with *λ*_em_ = 714 nm and a small Δ*E*_ST_ of 0.172 eV based on calculation, implying the importance of further experimental research on heptazine derivatives.

## Conclusion and Outlook

In summary, we have provided an overview of monomeric and polymeric heptazine-based *π*-conjugated materials for light-emitting. The historical introduction of heptazine was meticulously described. Benefiting from the intriguing electronic, optical, thermal, and mechanical properties, heptazine-based materials have roused tremendous research interest in the field of light-emitting. The metal ion-containing, polymeric g-C_3_N_4_-based, monomeric heptazine-based light-emitting materials, and devices are systematically summarized, which is conductive to stimulate numerous efforts in the development of heptazine-based functional materials. By comparison, the number of heptazine-based light-emitting materials is much less than that of heptazine-based photocatalytic ones, although some heptazine derivatives have exhibited great potential in practical applications. Therefore, it could be anticipated that more high performance heptazine-based light-emitting materials and devices will be realized through elaborate molecular design in the near future.
